# Minimally Invasive Surgical Treatment of Pilonidal Disease: Mid-Term Retrospective Analysis of a Single Center

**DOI:** 10.3389/fped.2019.00215

**Published:** 2019-06-04

**Authors:** Ana Isabel Oliveira, Catarina Barroso, Angélica Osório, Jorge Correia-Pinto

**Affiliations:** ^1^Department of Pediatric Surgery, Hospital de Braga, Braga, Portugal; ^2^School of Medicine, Life and Health Sciences Research Institute (ICVS), University of Minho, Braga, Portugal; ^3^ICVS/3B's - PT Government Associate Laboratory, Braga/Guimarães, Portugal

**Keywords:** Sacrococcygeal Pilonidal Disease, minimally invasive technique, excision with primary closure, children and adolescents, relapse

## Abstract

**Background:** Sacrococcygeal Pilonidal Disease (PD) is commonly treated with excision and primary closure techniques (EPC). Minimally invasive techniques (MIT), such as EPSiT and *Pit-picking*, had been recently advocated promising better outcomes. We analyzed mid-term results from our center after introduction of MIT to treat PD.

**Methods:** Patients submitted to MIT (*n* = 44) with a median follow-up of 37 months were analyzed and compared with patients submitted to EPC (*n* = 70) with a median follow-up of 5 years. Both groups included patients operated in our department between 2011 and 2016 and have similar demographic and clinical characteristics. We compared operative time and post-operative parameters such as time with pain, dressing time and time to relapse.

**Results:** The post-operative time with pain was significantly lower, whereas the dressing time was significantly longer, in MIT when compared to the EPC group. The relapse rate was similar in both groups but the follow-up is shorter in the MIT group. In addition, the analysis of patients free of disease using Kaplan-Meier curves revealed that relapse tends to occur more precociously in MIT than in EPC patients (*p* = 0.014). Interestingly, in the subgroup of patients with previous surgery, MIT's relapse rate was significantly lower than in the EPC group (30 vs. 100%, *p* < 0.001).

**Conclusions:** MIT has the advantage of having a shorter time with pain in the postoperative period, while EPC benefits from a shorter dressing time. In general, the relapse of the disease tends to manifest more precociously in MIT patients. Moreover, in the subgroup of patients with previous surgery, MIT seems to have significantly better results when compared to EPC.

## Introduction

Sacrococcygeal Pilonidal Disease (PD) is an acute inflammatory disease that mainly affects the sacrococcygeal region (1). It is defined by a midline sinus filled with hair, which can infect and lead to an abscess and/or fistula to the skin. The symptoms are commonly pain and drainage of purulent or hemorrhagic fluid (1, 2). It is associated with great morbidity and has great impact on the quality of life (3). Most patients are younger than 25 years of age at the inaugural episode (1, 2, 4). It is often related to obesity, sedentarism, and family history. Hairy individuals with increased sweating, thick skin and deep gluteal clefts are more prone (1, 2, 4). Contrary to what one might think, personal hygiene does not seem to be involved (2).

There is still no consensus regarding the best treatment and various surgical techniques have been described (1–4). In cases where the abscess is the initial presentation, acute-phase drainage can be the definite treatment (1, 5) For the remaining, the ideal method would be the one with the lowest morbidity, shorter healing time and lower relapse rate (1–3). Fostering this, surgeons propose different techniques from lay open, excision with primary closure to minimally invasive techniques (MIT).

The classic excision with primary closure (EPC) consists of an elliptical cutaneous incision including the openings of the fistulas and excision of the cyst in depth, followed by approximation of the tissues by planes and cutaneous suture (2). Some authors suggest that techniques using suture line lateral to the natal cleft while flattening it (such as Karydakis, Rhomboid excision, and Limberg flap reconstruction) cause less suture dehiscence and have lower relapse rates than techniques with suture in the midline within the natal cleft (3, 6). Even so, pediatric surgeons commonly use EPC with suture in the midline because it seems to have better cosmetic results.

Within minimally invasive techniques, the endoscopic pilonidal sinus treatment (EPSiT) allows a direct and magnified visualization of the cavity and fistulous trajectories, which theoretically limits the probability of residual disease and provides a better hemostasis (7–9). Whereas the *Pit-picking* is a similar technique, but without using the magnified visualization—the fistulous trajectories are removed using a biopsy punch, followed by curettage and washing (10). In both, there is minimal tissue excision and the wound is left open (7–10).

The relapse rate of PD can occur in excess of one third of cases, with the great majority of all recurrences concentrated within the first 5 years from surgery (3, 11). Risk factors for relapse are not consensual in children. Commonly, authors suggest that prolonged infection, excessive hair growth or dehiscence increases the propensity to relapse in adults (1). Contra-intuitively, pre-, peri-, or post-operative antibiotherapy is not associated with a lower relapse rate and thus should be limited for infected PD. Laser hair removal has been associated with a lower relapse rate (12–16). Nevertheless, it is accepted that relapse is time-dependent, supporting the idea of considering 5 years of follow-up as the benchmarking (11).

During the last years, MIT had been advocated promising better outcomes (7, 9, 17–19). This led us to introduce MIT in our department since the last 3 years. Herein, we analyzed mid-term results from our center after introduction of MIT to treat PD.

## Materials and Methods

### Study Design and Methods

An observational, descriptive and retrospective analytical study was performed. One hundred and fourteen patients were submitted to surgical treatment of Sacrococcygeal Pilonidal Disease (PD) in our department between 2011 and 2016. We gathered data through consultation of the clinical registry and telephone interviews with the patients, after obtaining the informed consent. Patients that were already discharged from outpatient visits were interviewed by phone and re-evaluated by a surgeon whenever a relapse was suspected.

Patients were divided into two groups according to the surgical technique used: (i) MIT group, where patients were operated by EPSiT or *Pit-Picking* techniques; (ii) EPC group, where patients were submitted to EPC in the midline. Inclusion criteria were as follows: Patients with the diagnosis of PD submitted to surgery in our Pediatric Surgery Department with a follow-up time of at least 2 years. Patients with more than one surgery during the study period were only included once with data corresponding to the last surgery. Six patients were excluded once they could not respond to the telephone interview. Relapse was determined: (i) by the need for a new surgery; or (ii) if diagnosed by a pediatric surgeon and registered in the clinical chart.

The studied variables were: Age (years); Gender; Body Mass Index (BMI); Presence of fistulas; Location of the fistulas; Disease onset time (months); Previous surgery; Family History; Operating time (minutes); Intra-operative Antibiotherapy; Time with pain (days); Time with analgesics (days); Time to return to daily life activity (days); Time to walk without pain (days); Time to sit without pain (days); Dressing time (days); Post-operative complications; Depilation; Relapse; Time to relapse (months); Follow-up time (months).

Additionally, the study compared MIT and EPC in smaller groups according to the evolution and presentation of the disease (previous surgeries: yes or no; fistulas: none, single or multiple; disease onset time: less than 6 months, 6 to 12 months, or more than 12 months). Finally, yet importantly, we analyzed possible factors that could contribute to relapse: Age; Gender; BMI; Presence of fistulas; Disease onset time (months); Previous surgery; Family History; Intra-operative Antibiotherapy; Dressing time (days); Post-operative complications (i.e., infection and abscess); Depilation.

### Surgical Technique

In the EPC, an elliptical cutaneous incision was performed, including the openings of the fistulas; the sinus was excised in depth, followed by approximation of the tissues and cutaneous suturing in the midline (1, 3).

EPSiT included a small circular incision of 0.5 cm around the fistula opening to insert the fistuloscope as developed by Meinero in 2011. Hair removal, cleaning of the infected area and cauterization of the sinus granulation tissue and fistulas were performed (7).

*Pit-Picking* consisted on the excision of the fistulas and curettage as described by Bascom in 1980 (10).

### Ethical Considerations

This study was approved by the Ethical Committee of our Hospital (Reference 103/2017) and, after that, approved by the Ethical Subcommittee of Life and Health Sciences of University of Minho.

### Statistics

Normality was tested by graphical methods (histograms, boxplots, Q-Q plots) numerical methods (skewness and kurtosis indices) and the Shapiro-Wilk test. In cases where the sample was significantly different from a normal distribution (age, disease onset time, time with pain, time with analgesics, time to return to daily life activity, time to walk without pain, time to sit without pain, dressing time and follow-up time) a non-parametric test, Mann-Whitney U-test, was used to compare both groups. In the remaining cases, the independent sample *T*-Test was used.

The chi-square test was also used to compare the categorical variables. A survival analysis was performed to show the time to relapse on a Kaplan-Meier curve. The Log Rank test was used to compare the time to relapse between the two groups. Finally, a Logistic Regression was used to analyze possible factors that could contribute to relapse. The statistical analysis was done using the IBM SPSS Statistics for Windows, Version 25.0. Significant results were considered for *p* < 0.05.

## Results

A total of 114 surgeries were performed, 44 by MIT and 70 by EPC. The characteristics of the groups are shown in Table 1. There were no significant differences between the two groups regarding gender, BMI, location of the fistulas, disease onset time, existence of previous surgery (relapsed disease) and family history, but a significantly higher number of MIT patients presented multiple fistulas.

**Table 1:** Clinical characterization of the groups.

	**MIT**	**EPC**	***p*-value**	**Effect size**
Age (years), Mdn (IQR)	16.0 (2)	15.0 (2)	***p*** **= 0.001**	***r*** **= −0.3**
**Gender**			*p* = 0.959	*phi* = −0.005
Male, *n* (%)	31 (70.5%)	49 (70%)		
Female, *n* (%)	13 (29.5%)	21 (30%)		
**BMI classification**			*p* = 0.525	*phi* = 0.164
Thin, *n* (%)	0 (0%)	1 (2.2%)	
Normal weight, *n* (%)	18 (47.4%)	23 (51.1%)	
Overweight, *n* (%)	9 (23.7%)	13 (28.9%)		
Obese, *n* (%)	11 (28.9%) (*n* = 38)	8 (17.8%) (*n* = 45)		
**Number of fistulas**			***p*** **= 0.002**	***phi*** **= 0.402**
None, *n* (%)	2 (4.5%)	14 (30.4%)	
Single, *n* (%)	12 (27.3%)	15 (32.6%)		
Multiple, *n* (%)	30 (68.2%) (*n* = 44)	17 (37%) (*n* = 46)	
**Localization of fistulas**			*p* = 0.452	*phi* = −0.090
Median line, *n* (%)	33 (78.6%)	24 (85.7%)	
Mid-lateral, *n* (%)	9 (21.4%) (*n* = 42)	4 (14.3%) (*n* = 28)		
Disease onset time (months), Mdn (IQR)	6.50 (9) (*n* = 42)	5.00 (7) (*n* = 64)	*p = 0*.096	*r* = −0.16
Previous surgeries, *n* (%)	10 (22.7%)	10 (14.3%)	*p* = 0.249	*phi* = −0.108

*MIT, Minimally Invasive Technique; EPC, Excision with Primary Closure; Mdn, median; IQR, interquartile range; n, number of patients. For some parameters, we could only get the information from the number of patients presented within brackets. The bold values represent statistically significant results*.

As shown on Table 2, the duration of the operation for MIT was significantly shorter as well as the duration of post-operative pain. On the other hand, EPC had significantly shorter dressing time. In addition, the time with analgesics (median = 1 day) and the necessary time to return to daily life activity (median = 3 days), walk without pain (median = 1 day) and sit without pain (median = 1 day) were consistently shorter on MIT than in EPC (*p* < 0.001). It is important to highlight that we did not find differences between both groups regarding the use of intra-operatory antibiotherapy, post-operative complications and depilation of the sacrococcygeal zone.

**Table 2:** Comparative analysis of the intra- and post-operative parameters.

	**MIT**	**EPC**	***p*-value**	**Effect size**
Operating time (min), (*M ± SD*)	36.1 ± 14.9 (*n* = 44)	48.7 ± 16.4 (*n* = 69)	***p < 0.001***	***g*** **= −0.793**
Time with pain (days), Mdn (IQR)	0 (3) (*n* = 38)	8 (28) (*n* = 43)	***p < 0.001***	***r*** **= −0.49**
Dressing time (days), Mdn (IQR)	30.0 (21) (*n* = 41)	15.0 (20) (*n* = 41)	***p = 0.001***	***r*** **= −0.36**

*MIT, Minimally Invasive Technique; EPC, Excision with Primary Closure; M ± SD, mean ± standard deviation; Mdn, median; IQR, interquartile range; n, number of patients. For some parameters, we could only get the information from the number of patients presented within brackets. The bold values represent statistically significant results*.

**Figure 1:**
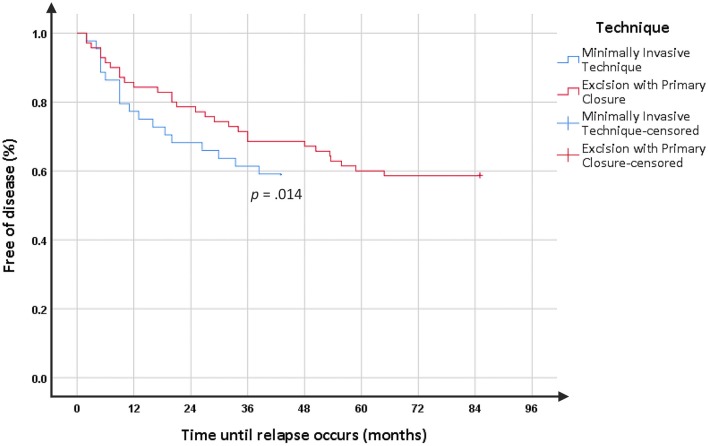
Kaplan Meier's curve of time to relapse.

The 5-year relapse rate from our department using EPC was 41.4%, whereas the mid-term relapse rate of MIT was 40.9%. However, it should be emphasized that the follow-up time was shorter in MIT than in EPC (37 vs. 55 months, *p* < 0.001). In fact, the analysis of patients free of disease using Kaplan-Meier curves (Figure 1) revealed that relapse tends to occur more precociously in MIT than in EPC patients (14.5 months vs. 26 months, *p* = 0.014). Interestingly, in the subgroup of PD patients with previous surgery (*n* = 10 in each group), MIT relapse rate was significantly lower than in the EPC group (30 vs. 100%, *p* < 0.001). Other subgroups' analysis (number of fistulas and time of disease) did not introduce differences between MIT and EPC (*p* > 0.05).

Regarding the factors that contribute to relapse, the logistic regression identified only the variable “post-operative complications” with a significant result (*p* = 0.037; *r*^2^ = 0.052).

## Discussion

This study confirms that MIT as treatment of PD in adolescents significantly attenuate post-operative discomfort, which would already be expected according to the literature (7, 8). However, the mid-term analysis suggests that they do not offer a lower relapse rate when compared to the classic excision and primary closure technique. Surprisingly, in the subgroup of patients with previous surgery, MIT seem to overcome all results obtained with the excision and primary closure technique.

Overall, this study compared two groups with similar pre-operative characteristics except for age and number of fistulas but these had low effect size (20). In addition, this may reflect a preference of the surgeon for MIT when there are multiple fistulas (avoiding a wider excision). That difference can also explain, at least in part, the longer dressing and healing time in the MIT group, as more fistulas are associated with a longer dressing time (7).

Early post-operative morbidity should be taken into special consideration as it greatly interferes with patients' quality of life, especially when postoperative complications such as dehiscence occur. As many others, this study shows that EPSiT improves psychological state, social function and pain (7, 19, 21, 22). *Pit-picking* is reported to have the same results, like it was expected, once EPSiT only complements *Pit-picking* with the endoscopic view (23).

Introduction of MIT created expectations that the relapse rate would be reduced. However, the great majority of studies reporting this results in children have < 1–2 years of follow-up (17, 18, 24). Our study clearly demonstrates that relapse is more dependent on time than operative technique, which explains the variability in the relapse rates we can find in the literature, likely because it depends on the time elapsed from surgery in the study (3, 11). In a recent analysis on 583 young adults with a 20-year follow-up, Doll et al. found a 44% relapse rate of primary midline closure (25). In addition, studies show that the great majority of relapses occurs within 5 years from surgery (3, 11), because of that we compared our MIT group with our benchmark represented by the EPC group with a median follow-up of 5 years. Our study even suggests that the relapse might be more precocious in MIT, raising the possibility of a higher relapse rate as time goes onward.

According to the literature, MIT can be used for both first surgery or relapsed PD, being equally effective (7). In this study, we verified some interesting results, as in relapsed cases MIT presented better results. This was not mentioned by any other study up to now. Although our series with previous surgery is small, these results are worth further investigation.

Possible causes to relapse include tracts not completely excised associated with newly perforating hairs into the healing pilonidal skin scar (3). However, many factors are still not understood. Many studies tried to identify risk factors like prolonged sitting job, familiar and personal history, longer cavity diameter, young age and high BMI but none of them studied a pediatric population (26–28). In our study, we did not find a definitive risk factor as post-operative complications only predicted 5% of the relapses.

Limited by being a retrospective, single-center study, our work has the advantages of using a population instead of a sample (thus avoiding a selection bias); including more than 100 patients which allows to draw safer conclusions; having homogeneous groups; the groups' characteristics being accordingly to what is described in the literature i.e., mostly males, overweight/obese, and young people with familiar background (1, 2, 4).

As far as we know, our study provides the longest follow-up of MIT in children. Knowing that the PD relapse is essentially a time-dependent phenomenon, it is imperative to increase the follow-up time, especially in the MIT group, in order to draw definitive conclusions. Only then, we can verify our premise that MIT patients relapse more precociously and if the relapse rate is equal or lower whilst with a different relapse pattern. In addition, MIT might have an important role in relapsed cases if the results observed in this study are confirmed.

## Conclusion

MIT has the advantage of having a shorter time with pain in the postoperative period, while EPC benefits from a shorter dressing time. In general, the relapse of the disease tends to manifest more precociously in MIT patients. Moreover, in the subgroup of patients with previous surgery, MIT seems to have significantly better results when compared to EPC.

## Ethics Statement

This study was carried out in accordance with the recommendations of the Ethical Committee of Hospital of Braga with written informed consent from all subjects. All subjects gave written informed consent in accordance with the Declaration of Helsinki. The protocol was approved by the Ethical Committee of our Hospital (Reference 103/2017) and, after that, approved by the Ethical Subcommittee of Life and Health Sciences of University of Minho.

## Author Contributions

Study conception was performed by AIO, CB, AO, and JC-P and data acquisition by AIO. AIO, CB, AO, and JC-P were involved in interpretation and analysis of data. The manuscript was written by AIO and revised by CB, AO, and JC-P.

### Conflict of Interest Statement

The authors declare that the research was conducted in the absence of any commercial or financial relationships that could be construed as a potential conflict of interest.
